# Le surpoids, l’obésité et le contrôle glycémique chez les diabétiques du centre de référence provincial de diabète (CRD), Kénitra, Maroc

**DOI:** 10.11604/pamj.2017.27.189.9535

**Published:** 2017-07-11

**Authors:** Zeghari Lotfi, Youssef Aboussaleh, Rachid Sbaibi, Imane Achouri, Rachid Benguedour

**Affiliations:** 1 Laboratoire de Nutrition, Santé & Environnement, Département de Biologie, Faculté des Sciences, Université Ibn Tofail BP 133, Kénitra 14 000, Maroc; 2 Laboratoire Biochimie, Biotechnologie, Santé et Environnement, Département de Biologie, Faculté des Sciences, Université Ibn Tofail, BP 133, Kénitra 14 000, Maroc

**Keywords:** Diabète, prévalence, IMC, surpoids, Hémoglobine glycosylée, glycémie à jeun, Diabetes, prevalence, BMI, overweight, glycosylated hemoglobin, fasting blood glucose

## Abstract

**Introduction:**

Le diabète est définit comme un trouble de l'assimilation, de l'utilisation et du stockage des sucres apportés par l'alimentation, sa prise en charge est assurée par le suivi du surpoids et l'obésité et le contrôle glycémique régulier. L'objectif de ce travail était l'étude du surpoids, l'obésité et le contrôle glycémique chez 2227 diabétiques de différent type (type 1, 2 et gestationnel), consultants le centre de référence provincial de diabète (CRD), Kénitra-Maroc.

**Méthodes:**

L'étude s'est déroulée sur une période d'une année du mois janvier au mois décembre 2015, L'évaluation du surpoids et l'obésité a été effectuée par le calcul de l'Indice de Masse Corporelle (IMC=Poids/Taille^2^ (Kg/m^2^)), elles sont définit respectivement par IMC > 25 Kg/m^2^, et IMC > 30 Kg/m^2^, le poids et la taille ont été mesurés selon les recommandations de l'organisation mondiale de santé (OMS), Le contrôle glycémique a été effectué par l'analyse sanguine de l'Hémoglobine glycosylée et de la Glycémie à jeun. Les normes sont 7% pour l'Hémoglobine glycosylée et 0,70g/l à 1,10g/l pour la Glycémie à jeun.

**Résultats:**

L'intervalle d'âges des patients est compris entre 8 mois et 80 ans, avec une dominance des diabétiques provenant du milieu urbain (74%) par rapport à ceux provenant du milieu rural (26%). Le surpoids touche l'ensemble de cette population. L'IMC moyen des femmes tends vers l'obésité (IMC≈30): (29,21 Kg/m^2^ ± 3,1) pour le diabète gestationnel et (29,15 Kg/m^2^ ± 3,2) pour le diabète de type 2. Les valeurs du contrôle glycémique sont supérieures aux normes: avec 8,5% ± 2,6 > 7% pour l'hémoglobine glycosylée et 1,5 g/l ± 1,3 > 1,10g/l pour la Glycémie à jeun. La différence entre les valeurs de l'hémoglobine glycosylée entre les hommes (8,5 7% ± 2,6) et les femmes (8,1% ± 2,3) n'est pas significative (P > 0,05), même chose pour la Glycémie capillaire à jeun: pour les hommes (1,44 g/l ± 1,1) et les femmes (1,43 g/l ± 1,2). Les coefficients de corrélation de Pearson sont hautement significatifs (P<0,005); d'une part entre IMC et la Glycémie à jeun (r = 0,5) et d'autre part entre IMC et les valeurs de l'Hémoglobine glycosylée (r = 0,4).

**Conclusion:**

L'ensemble des diabétiques présente des valeurs de l'IMC et du contrôle glycémique, supérieures aux normes. Des recherches approfondies sont nécessaires sur ces diabétiques afin de dresser un programme urgent de remédiation.

## Abstract (en)

**Introduction:**

Diabetes is a disorder of assimilation, use and storage of sugars provided in the diet. Its management is based on follow-up of overweight and obese patients and on regular glycemic control. This study aimed to analyze overweight, obesity and glycemic control in 2227 patients with different types of diabetes (type 1, 2 and gestational) presenting to the Provincial referral center of diabetes (RCD) in Kenitra, Morocco.

**Methods:**

We conducted a study over the period January-December 2015. Overweight and obesity assessment was performed using Body Mass Index calculator (BMI = weight/height^2^ (kg/m^2^). Overweight and obesity were defined by BMI > 25 kg/m^2^and BMI > 30 kg/m^2^ respectively; the weight and the height were measured according to World Health Organization's recommendations. Glycemic control was based on glycated hemoglobin levels and fasting blood glucose test. Current guidelines recommend a glycosylated hemoglobin level of 7% and a fasting blood glucose of 0.70g/l - 1.10g/L.

**Results:**

The age of patients ranged from 8 months to 80 years, with a prevalence of diabetic patients from the urban environment (74%) compared to those from the rural areas (26%). The entire study population was overweight. The average BMI of women showed a trend toward obesity (BMI≈30): (29.21 kg/m^2^ ± 3,1) in patients with gestational diabetes and (29.15 kg/m^2^ ± 3.2) in patients with type 2 diabetes. Blood sugar levels were above the standards: 8.5% ± 2.6 > 7% for glycosylated hemoglobin and 1.5 g/L ± 1.3>1.10g/L for fasting blood glucose. The difference between glycosylated hemoglobin levels between men (8.57% ± 2.6) and women (8.1% ± 2.3) were not significant (p > 0.05), it was the same with fasting blood glucose: men (1.44 g/L ± 1,1) and women (1.43 g/L ± 1.2). Pearson's correlation coefficients were highly significant (p<0.005); on the one hand between BMI and fasting blood glucose(r = 0.5) and on the other hand between BMI and glycosylated hemoglobin levels (r = 0.4).

**Conclusion:**

The entire study population had BMI and glycaemic control levels above the standards. More research is needed on diabetic patients in order to develop a remediation plan.

## Introduction

Le diabète est un problème majeur de santé publique par sa prévalence croissante et son impact socio-économique [1]. Selon les estimations de l'OMS [2], plus de 356 millions de personnes dans le monde sont atteintes de diabète, ce chiffre risque d'être multiplié par deux vers l'année 2030. Au Maroc, plusieurs études ont montré que la prévalence du diabète est de 6,6%, soit plus d'un million et demi de Marocains en 2010; selon JE. Shaw et al en 2010 [3] ce chiffre atteindra 2,5 millions à l'horizon de 2030. Le diabète par définition peut être regroupé en deux types majeurs: le diabète de type 1 appelé aussi diabète insulino-dépendant qui est causé par la destruction des cellules bêta du pancréas, d'où l'incapacité de la personne atteinte à sécréter de l'insuline [4]; le diabète de type 2 appelé aussi diabète sucré, qui se caractérise par une résistance à l'insuline et qui se traduit par l'élévation chronique de la concentration de glucose dans le sang (hyperglycémie) [5]. En effet, il existe d'autres formes de diabète, comme le diabète gestationnel qui peut être transitoire et lié à une résistance à l'insuline pendant la grossesse mais peut également persister après la grossesse. [6]. Dans la majorité des cas, le diabète est associé à un ensemble d'anomalies regroupé sous le vocable de syndrome métabolique qui représente un risque majeur de morbidité [7, 8]. Le diabète est incurable, l'absence d'un traitement efficace peut causer différentes complications: à savoir la rétinopathie; la néphropathie, les cardiopathies et les amputations, d'où la nécessité d'un traitement à vie, par un contrôle glycémique, qui améliora la qualité de vie des patients et en même temps réduira le coût élevé de la prise en charge pour le secteur de la santé, dont la dépense au Maroc, en 2010, a dépassé les 206 millions USD [9]. D'autant plus, le surpoids et l'obésité sont considérés comme des facteurs de risque essentiel du diabète, leur contrôle par une alimentation équilibrée comblée avec une activité physique régulière permet de prévenir cette maladie [10, 11]. Le présent travail vise l'étude du surpoids et l'obésité et le contrôle glycémique chez les diabétiques du seul centre de référence provincial de diabète (CRD) à kénitra, Maroc.

## Méthodes

La présente étude a été effectuée à kénitra dont la population totale a été estimée de 1034114 habitants en 2014 [12], dont 600963 en milieu urbain et 433151 en milieu rural. L'échantillon étudiée est constituée de 2227 diabétiques (58% des Femmes; 42% des hommes), qui ont consultés le CRD à kénitra du mois janvier au mois décembre 2015, l'effectif des patients venant de l'urbain domine avec un pourcentage de 74%. L'intervalle d'âges est de 8 mois à 80 ans. Les données ont étés collectées au moyen d'un questionnaire contenant les informations sociodémographiques des patients. Le surpoids et l'obésité ont été déterminés par le calcul de l'Indice de Masse Corporelle (IMC = Poids/Taille^2^), (Kg/ m^2^)),qui sont définit respectivement par IMC > 25 Kg/ m^2^) et IMC > 30 Kg/ m^2^). Les mesures du poids et de la taille sont effectuées selon la norme standard de l'OMS [13], en vêtement d'intérieur, sans chaussure. Le poids a été obtenus à l'aide d'une pèse personne mécanique de marque Seca 761 - Classe IIII, avec une précision de 0,1kg. La taille a été mesurée à l'aide d'une toise avec une précision de 0,1 cm. Le contrôle glycémique a été effectué par deux analyses sanguines jumelles de la glycémie: l'hémoglobine glycosylée (L'HbA1C) qui permet d'évaluer l'équilibre glycémique durant les deux à trois mois précédents; c'est un bon indicateur du sucrage de notre organisme et donc du risque de complications diabétiques, et la Glycémie capillaire à jeun, qu'est un instantané de l'état glycémique. Les normes sont 7% pour l'hémoglobine glycosylée et 0,70g/l à 1,10g/l pour la Glycémie à jeun. La Glycémie capillaire à jeun a été mesurée par un lecteur de glycémie de marque Accu-Chek Active par l'analyse d'une goutte de sang, prélevée au bout du doigt, à l'aide d'un stylo auto-piqueur. L'hémoglobine glycosylée a été mesurée par un appareil de marque «SIEMENS», qui permet le dosage de l'hémoglobine glycolysée à partir d'une goutte de sang par l'intervention des cassettes réactives, fourni par le ministère de la santé. Les données ont été saisies et analysées sur logiciel SPSS version 16. Les fréquences et les pourcentages ont été calculés pour les variables qualitatives et les moyennes et écarts types (δ) pour les variables quantitatives. Avant l'inclusion dans l'étude, l'autorisation de l'enquête est acquise auprès de la délégation provinciale de la santé, le personnel du (CRD) et les patients ont été informés sur les objectifs de l'enquête. Le consentement oral des patients a été obtenu avant l'administration du questionnaire, par ailleurs, l'anonymat et le respect de la confidentialité des données ont été assurés.

## Résultats

L'échantillon de la présente étude est de 2227 patients (58% des Femmes; 42% des hommes), avec une dominance des patients venant de l'urbain (74%).

**Le surpoids et l'obésité:** Le **Tableau 1** représente les prévalences des diabétiques et les IMC moyens de l'échantillon selon le sexe et le type du diabète. Le diabète de type 2 représente 88% des patients, 11% pour le diabète de type1 et seul 1% pour le diabète gestationnel. Nous remarquons que le surpoids touche l'ensemble des patients (IMC>25 Kg/ m^2^), il est plus marqué chez les femmes (IMC>29,15 >25 Kg/m^2^) que les hommes (IMC>27,78 ± 2,3Kg/ m^2^). L'IMC moyen des femmes tends vers l'obésité (IMC≈30): (29,21 Kg/ m^2^ ± 3,1) pour le diabète gestationnel et (29,15 Kg/ m^2^ ± 3,2) pour le diabète de type 2.

**Tableau 1:** Prévalence du diabète et les IMC moyens de l’échantillon selon le sexe et le type du diabète

Type de diabète	Sexe	Ages (Moy± δ)	IMC (Moy± δ)	Prévalence dans le type (%)	Prévalence total (%)
Type 1					11%
	Femme	15,3±4,1	………	44%	
	Homme	12,1±5,3	………	56%	
Type 2					88%
	Femme	45,0±2,3	29,15± 3,2	57%	
	Homme	50,2±1,8	27,78 ± 2,3	43%	
Gestationnel	Femme	40,2±7,5	29,21 ± 3,1	100%	1%

**Figure 1:**
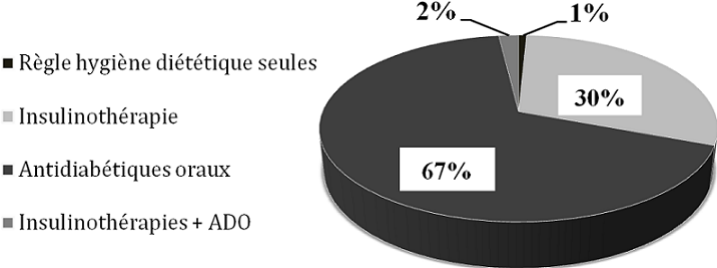
Répartition de l’échantillon selon le type de traitement utilisé

**Le contrôle glycémique:** Le **Tableau 2** rapporte des valeurs du contrôle glycémique supérieures aux normes: avec 8,5% ± 2,6 > 7% pour l'Hémoglobine glycosylée et 1,5 g/l ± 1,3 > 1,10g/l pour la Glycémie capillaire à jeun. La différence entre les valeurs de l'Hémoglobine glycosylée entre les hommes (8,5 7% ± 2,6) et les femmes (8,1% ± 2,3) n'est pas significative (P > 0,05), idem, pour la Glycémie capillaire à jeun: pour les hommes (1,44 g/l ± 1,1) et les femmes (1,43 g/l ± 1,2). Les coefficients de corrélation de Pearson sont hautement significatifs (P < 0,00); d'une part entre IMC et la Glycémie capillaire à jeun(r = 0,5) et d'autre part entre IMC et les valeurs de l'Hémoglobine glycosylée (r = 0,4). La Figure 1 montre quatre types de traitement utilisé par ces diabétiques: les règles hygieno-diététiques qui s'élucident dans l'alimentation équilibré, les antidiabétiques oraux (ADO) qui ont une action modulatrice du l'effet de l'insuline dans l'organisme, les ADO+ insuline et l'insuline seule, sont destiné aux patients insulino-dépendant, le but de ces traitements chez les diabétiques est en général le maintien des limites normales de la glycémie afin d'éviter le développement des complications aiguës [14–16]. On remarque que les antidiabétiques oraux (ADO) est le traitement le plus fréquent de cet échantillon avec un pourcentage de 67%.

**Tableau 2:** Le bilan du contrôle glycémique par analyses sanguines des diabétiques

Analyses sanguines	Homme	Femme	Valeur de P
Hémoglobine glycosylée	8,5% ± 2,6	8,1% ± 2,3	P> 05; NS
Glycémie capillaire à jeun g/l	1,44 g/l ± 1,1	1,43 g/l ± 1,2	P> 05; NS

NS = non significatif; p > 0,05

## Discussion

Les diabétiques de la présente étude venant de l'urbain dominent avec un pourcentage de 74%; ce pourcentage est expliqué par le nombre élevé de la population en milieu urbain 600963(60%) que le rural 433151 (40%) dans la province de kénitra [12]. 88% des diabétiques sont de type 2, ce résultat est normal car ce type est la forme de diabète la plus commune dans le monde, avec une prévalence de 90 à 95% [17–19]. 11% des diabétiques sont de type1, ce résultat est proche de 5 à 10% trouvé dans le monde [20], et seul 1% correspond au diabète gestationnel, cette prévalence est similaire à 4% mentionné par des études populationnelles canadiennes [21, 22]. Le surpoids touche l'ensemble de cette population (IMC > 25 Kg/m^2^), il est plus marqué chez les femmes (IMC > 29,15 >25 Kg/ m^2^) que les hommes (IMC > 27,78 ± 2,3Kg/ m^2^) cette différence est la même rapportée par l'enquête national sur l'anthropométrie en 2011 au Maroc ou le surpoids est plus fréquent chez les femmes (61,1%) que chez les hommes (38.9%) [23]. L'IMC moyen des femmes tends vers l'obésité (IMC ≈ 30): (29,21 Kg/ m^2^ ± 3,1) pour le diabète gestationnel et (29,15 Kg/ m^2^± 3,2) pour le diabète de type 2; ces résultats sont semblables à ceux rapportés par Nthangeni et coll. en 2002 [24] et Alebiosu et Odusan en 2004 [25]; et selon Rooney et Schauberger en 2002 [26]; et gore et coll.; en 2003 [27] la présence du surpoids chez les femmes avec un diabète gestationnel a été expliqué, par le gain de poids excessif pendant la grossesse ( il est de 0,5 à 3 kilogrammes et peut même atteindre jusqu'à 17,7 kg chez certaines femmes); et la rétention de de cet excès de poids persiste après l'accouchement [27].

Les valeurs du contrôle glycémique de ces patients sont supérieures aux normes de [16, 28, 29]: avec 8,5% ± 2,6 > 7% pour l'hémoglobine glycosylée et 1,5 g/l ± 1,3 > 1,10g/l pour la glycémie capillaire à jeun; des résultats semblables ont été approuvés par l'étude de A. coulibaly et al en 2007 [30], qui ont travaillé sur des diabétiques de type 2 et qui ont trouvé une valeur d'Hémoglobine glycosylée égale à 8,4 ± 2,3 et 1,44 ± 0,9 pour la glycémie à jeun. La différence entre les valeurs de Hémoglobine glycosylée entre les hommes (8,5 7% ± 2,6) et les femmes (8,1% ± 2,3) n'est pas significative (P > 0,05), idem, pour la Glycémie capillaire à jeun: pour les hommes (1,44 g/l ± 1,1) et les femmes (1,43 g/l ± 1,2), même résultat rapporté par l'étude de A. coulibaly et al en 2007 [30], ceci justifie que le diabète n'est pas lié au sexe. Les coefficients de corrélation de Pearson sont hautement significatifs (P < 0,005); d'une part entre IMC et la Glycémie à jeun (r = 0,5) et d'autre part entre IMC et les valeurs de l'Hémoglobine glycosylée (r = 0,4); ce résultat pourra être expliqué par le fait que le surpoids pourra conduire dans la majorité des cas à un diabète mal équilibré [31]. Les antidiabétiques oraux (ADO) est le traitement le plus fréquent avec un pourcentage de 67%; ce résultat est en lien directe avec le taux élevé des diabétiques de type 2 (88%), du fait que les ADO représentent le traitement utilisé dans la majorité des cas, pour traiter les diabétiques de type 2 [32]. 1% des patients est sous règles hygiéno-diététiques seules, et ceci pourrait être expliqué par le fait que dans la majorité des cas, le diabétique consulte le CRD en état d'hyper glycémie chronique où les règles hygiéno-diététiques: tel que l'alimentation équilibrée seule ne donnera pas les résultats désirés.

## Conclusion

L'ensemble de ces diabétiques présentent des valeurs de l'IMC et du contrôle glycémique, supérieures aux normes. Des recherches approfondies sont nécessaires sur ces diabétiques afin de dresser un programme urgent de remédiation.

### Etat des connaissances actuelles sur le sujet

- Le diabète est un problème majeur de santé public; du fait que sa prévalence est en croissance;
- Le diabète est une maladie chronique, incurable, avec un risque de complications;
- Le diabète nécessite une bonne prise en charge qui s'élucide dans un traitement à vie, un suivi du surpoids et l'obésité et un contrôle glycémique régulier.

### Contribution de notre étude à la connaissance

- L'étude du surpoids et l'obésité et le contrôle glycémique chez des diabétiques de la province de Kénitra;
- L'étude approuve les résultats des autres études sur les liens qui existent entre le surpoids, l'obésité et l'équilibre glycémique chez les diabétiques en fonction du sexe;
- Des recherches approfondies sont nécessaires sur ces diabétiques pour dresser un programme urgent de remédiation.

## Conflits d’intérêts

Les auteurs ne déclarent aucun conflit d'intérêt.
